# Comprehensive analysis of the 9p21 region in neuroblastoma suggests a role for genes mapping to 9p21–23 in the biology of favourable stage 4 tumours

**DOI:** 10.1038/sj.bjc.6602094

**Published:** 2004-08-10

**Authors:** J Mora, M Alaminos, C de Torres, P Illei, J Qin, N-K V Cheung, W L Gerald

**Affiliations:** 1 Department of Molecular Pathology, Memorial Sloan-Kettering Cancer Center, New York 10021, NY, USA; 2 Department of Oncology, Hospital Sant Joan de Déu, 08950 Esplugues, Barcelona, Spain; 3 Department of Biostatistics, Memorial Sloan-Kettering Cancer Center, New York 10021, NY, USA; 4 Department of Pediatrics, Memorial Sloan-Kettering Cancer Center, New York 10021, NY, USA

**Keywords:** neuroblastoma, CDKN2A, MTAP, nuclear factor I-B3, microarrays

## Abstract

Chromosome 9p21 is frequently deleted in many cancers. Previous reports have indicated that 9p21 LOH is an uncommon finding in neuroblastoma (NB), a tumour of childhood. We have performed an extensive analysis of 9p21 and genes located in this region (cyclin-dependent kinase inhibitor 2A – *CDKN2A/*p16^INK4a^, *CDKN2A/*p14^ARF^, *CDKN2B*/p15^INK4b^, *MTAP*, *interferon α* and *β* cluster). LOH was detected in 16.4% of 177 NB. The SRO was identified between markers D9S1751 and D9S254, at 9p21–23, a region telomeric to the *CDKN2A* and *MTAP* genes. A significantly better overall and progression-free survival was detected in stage 4 patients displaying 9p21–23 LOH. Hemizygous deletion of the region harbouring the *CDKN2A* and *CDKN2B* loci was identified in two tumours by means of fluorescent *in situ* hybridisation and MTAP was present by immunostaining in all but one tumour analysed. The transcriptional profile of tumours with 9p21–23 LOH was compared to that of NB displaying normal 9p21–23 status by means of oligonucleotide microarrays. Four of the 363 probe sets downregulated in tumours with 9p21–23 LOH were encoded by genes mapping to 9p22–24. The only well-characterised transcript among them was *nuclear factor I-B3*. Our results suggest a role for genes located telomeric of 9p21 in good risk NB.

Neuroblastoma (NB) is a tumour in which many chromosomal abnormalities have been detected (Takita *et al*, 1995; Mora *et al*, 2002a). The 9p21 region has been found to be deleted in a wide range of malignancies (Chin *et al*, 1998) and it has also been reported to be altered in NB tumours, although at a low frequency (Marshall *et al*, 1997; Giordani *et al*, 2002; Mora *et al*, 2002b). Three loci in 9p21 have been implicated as tumour suppressor genes (TSG): cyclin-dependent kinase inhibitor 2A – *CDKN2A/*p16^INK4a^, *CDKN2A/*p14^ARF^ and *CDKN2B*/p15^INK4b^. The proteins p16^INK4a^ and p14^ARF^ have unique first exons (exon *1β* and *1α*, respectively), but share exons *2* and *3* and are translated in different reading frames (Kamb *et al*, 1994a; Quelle *et al*, 1995). p16^INK4a^ functions as a regulator of the G1/S-phase transition by inhibiting the activity of cyclin-dependent kinases CDK4 and CDK6. This hampers the phosphorylation of the retinoblastoma (Rb) protein, contributing to cell cycle arrest (Serrano *et al*, 1993). Although alterations of the *CDKN2A* gene have been reported in many malignancies, it is a rare event in neuroblastoma (NB) (Beltinger *et al*, 1995; Kawamata *et al*, 1996; Castresana *et al*, 1997; Takita *et al*, 1997; Iolascon *et al*, 1998). However, deregulation of the p16-CDK/cyclin D-pRb pathway has been described in NB (Diccianni *et al*, 1996; Easton *et al*, 1998) and, interestingly, contradictory results have been reported concerning the correlation between clinical outcome in NB and *CDKN2A/*p16^INK4a^ expression (Takita *et al*, 1998; Omura-Minamisawa *et al*, 2001).

p14^ARF^ regulates both the p53 and pRb pathways, by binding to and inhibiting the function of the proto-oncogene *mdm-2* (Pomerantz *et al*, 1998), thus preventing p53 degradation (Lundberg and Weinberg, 1999; Sharpless and DePinho, 1999). It also binds E2F-1 and inhibits its transcriptional activity (Eymin *et al*, 2001). Whereas *CDKN2A/*p16^INK4a^ mutation selectively inactivates the Rb pathway, deletion of the *CDKN2A* locus impairs both the Rb and p53 pathways. Deletion of the *CDKN2A* locus also frequently affects the *CDKN2B* locus, which encodes p15^INK4b^, an important mediator of the antiproliferative effect of TGF-*β* (Hannon and Beach, 1994).

Other genes implicated in cancer also map to 9p21: *MTAP* (methylthioadenosine phosphorylase), and *interferon (IFN) α* and *β* clusters. The *MTAP* gene resides approximately 100 kb telomeric of *CDKN2A* and is frequently codeleted with it (24, 25). It encodes an ubiquitous enzyme which is essential in methionine and purine metabolism and is frequently deficient in cancer cell lines (Kamatani *et al*, 1981) and in some malignancies (Fitchen *et al*, 1986; Schmid *et al*, 1998; Garcia-Castellano *et al*, 2002). To the best of our knowledge, there are no reports on *MTAP* gene alterations in NB.

The *IFN* gene cluster resides approximately 500–1000 kb in the telomeric direction from *CDKN2A* (Olopade *et al*, 1995). It consists of the *IFN-β*_*1*_ gene (*INFB1*) and at least 25 genes and pseudogenes for *INF-α* (*INFA*) and *INF-ω* (Diaz *et al*, 1988). *MTAP* is codeleted with a frequency of >85% in cell lines bearing *CDKN2A* deletions, whereas the *IFN* gene cluster is deleted in whole or in part in >50% of p16^INK4a^ -deficient cell lines (Zhang *et al*, 1996). Deletions of the *IFN* gene cluster have also been described in lung cancer (Olopade *et al*, 1993), acute lymphoblastic leukaemia (Diaz *et al*, 1990), acute lymphocytic leukaemia (Einhorn *et al*, 1990), glioma cell lines (James *et al*, 1993) and head and neck cancer (Lydiatt *et al*, 1998).

Homozygous deletion of the 9p21 locus occurs frequently in malignancies such as bladder carcinomas (Cairns *et al*, 1994), melanomas (Kamb *et al*, 1994b), and other carcinomas (Nobori *et al*, 1994). We and others have previously reported a low frequency of loss of heterozygosity (LOH) at 9p21 in NB (Takita *et al*, 1995; Castresana *et al*, 1997; Marshall *et al*, 1997; Easton *et al*, 1998; Mora *et al*, 2001a; Thompson *et al*, 2001). However, conflicting results exist on the correlation between LOH at 9p21 and prognosis of NB patients (Takita *et al*, 1997; Mora *et al*, 2001a).

In order to further analyse the role of 9p21 region in NB biology, we investigated the incidence of LOH at 9p21 and the status and expression of all known genes in the region in a well-characterised series of NB tumours.

## MATERIALS AND METHODS

### Samples and patients

Samples of 177 NB were obtained from patients who underwent surgery at Memorial Sloan-Kettering Cancer Center (MSKCC), New York. They included 11 stage 4s; 64 local-regional (LR) and 102 stage 4. Their clinical features have been described elsewhere (Mora *et al*, 2000; 2001b; 2002a; 2002b). Matched normal tissues (peripheral blood or bone marrow not affected by tumour) were also procured. The specimens were obtained in accordance with a protocol approved by the Memorial Hospital Institutional Review Board.

All cases were collected from 1987 to 1999 and selected only based on the availability of good quality normal and tumour specimen. Of the 64 LR cases, 44 were initially diagnosed at Memorial Sloan-Kettering Cancer Center and 20 referred at relapse. Eight referred patients had prior chemotherapy for their LR NB. Of the 102 stage 4 patients, 76 samples (74.5%) were obtained at the time of diagnosis prior to any chemotherapy and 26 (25.5%) were obtained at the time of the second-look surgery after induction chemotherapy. In all, 84 (82%) of the 102 patients were managed at MSKCC from diagnosis and were analysed separately. Five patients came to our institution at the time of relapse from other centres and were then treated with N6/N7 protocols; six patients came to our institution having responded to other induction regimens and were continued therapy based on N6/N7 protocols; and six patients were treated elsewhere and came to our institution for 3F8 antibody-based therapy.

### Allelic analysis

**Figure 1:**
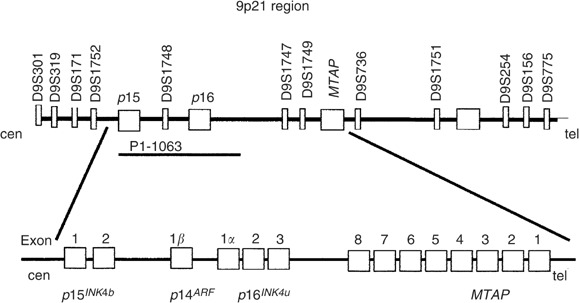
The 9p21 region. Genes located on it and some of the microsatellite markers used in the allelic analysis. The locus specific probe utilised for fluorescent *in situ* hybridisation is indicated with the name of the clone (P1 clone 1063, A Kamb, Myriad Genetics).

Allelic analysis for 9p21 was first evaluated in the complete series of 177 NB tumours using a set of four microsatellite markers (cent – D9S301, D9S319, D9S156 and D9S775 – pTer), according to methods previously described (Mora *et al*, 2000). Further allelotype analysis was carried out in cases showing LOH in the first screening, adding eight microsatellite markers (cent – D9S171, D9S1752, D9S1748, D9S1747, D9S1749, D9S736, D9S1751 and D9S254 – pTer). Primer sequences for polymorphic microsatellite loci were obtained from the Genome Data Base. The location of genes mapping to 9p21 and the microsatellite markers used in the analysis is depicted in Figure 1

.

### Interphase bicolour fluorescent *in situ* hybridisation (FISH)

The *CDKN2A* and *CDKN2B* genes copy number was analysed by interphase bicolour FISH in 21 of the 29 tumours displaying 9p21 LOH in the first allelotype screening. FISH was carried out on touch imprints as described in previous reports (Mora *et al*, 2001c), except for the probes. A locus-specific probe was used for detecting *CDKN2A/*p16^INK4a^, *CDKN2A/*p14^ARF^ and *CDKN2B*/p15^INK4b^ loss (P1 clone 1063, A Kamb, Myriad Genetics) and the chromosome 9 centromeric probe (CEP9 Vysis) was utilised as control. Control tissues were normal lymphocytes and the A673-Ewing sarcoma cell line with known homozygous *CDKN2A/*p16^INK4a^ deletion.

### PCR and sequencing of the *MTAP* gene

Exons 2, 4, 6 and 7 of the *MTAP* gene were analysed in 22 tumours, eight of them with LOH at 9p21 and 14 with normal 9p21 status. Genomic DNA was isolated using standard procedures. Polymerase chain reaction (PCR) and agarose gel electrophoresis were performed according to previously described methods (Garcia-Castellano *et al*, 2002).

### Immunohistochemistry for MTAP

Paraffin sections were obtained from 10 tumours in which LOH at 9p21–23 had been detected in order to evaluate MTAP protein expression. Immunohistochemistry was carried out as reported before (Garcia-Castellano *et al*, 2002). The anti-human MTAP chicken antibody was a kind gift of Dr Dennis Carson (UCSD Cancer Center). COS 7 cells were processed as positive controls and human osteosarcoma cell line HOS, U2OS and SaOS-2 as negative controls. Vessels and endothelial cells in each sample served as internal controls.

### Analysis of differentially expressed genes by oligonucleotide microarrays

Genome-wide expression profiles of seven NB displaying 9p21–23 LOH were compared to those of 17 NB with normal 9p21–23 status. Gene expression analysis was performed using Affymetrix Human Gene Array Set U95, which includes 63 175 features for individual gene/expressed sequence tags (ESTs) clusters, as described elsewhere (LaTulippe *et al*, 2002). Scanned image files were visually inspected for artefacts and analysed using Microarray Suite v5.0 (Affymetrix).

### Statistical analysis

To examine the association between allelic loss in 9p21 region and factors such as sex, ploidy, MYCN, age, ferritin and LDH at diagnosis, Fisher's exact test was performed and two-sided *P*-values were computed. The association between progression-free survival (PFS), defined as relapse, and overall survival (OS), defined as the time to death or last follow-up and clinicobiological variables, was assessed using the log-rank test (Cox and Oakes, 1984). Those factors which were potentially predictive of PFS and OS were entered into a multivariate analysis using the Cox proportional hazards model. Survival curves were generated using the method of Kaplan and Meier (Kaplan and Meier, 1958). All statistical calculations were performed using S-Plus 2000 (Mathsoft Inc. Seattle, Washington, USA).

Microarray expression data set was filtered to include only those probe sets detecting transcripts with mean expression values that differed by at least two-fold between the group with 9p21–23 LOH and the group without the allelic loss. Probes were then ranked based on the relative magnitude of the difference (*t*-test) between the two-sample sets. A transcript was considered to be downregulated in the 9p21–23 LOH group when the mean of fluorescent intensities for that particular mRNA was at least two-fold higher in the cohort with normal 9p21–23 status, and when the comparison of means provided a significant difference (*t*-test, *P*<0.01). We also utilised these parameters to compare the levels of expression of *CDKN2A/*p16^INK4a^, *CDKN2A/*p14^ARF^, *CDKN2B*/p15^INK4b^, *MTAP*, *IFNA* and *IFNB* mRNAs in both groups.

## RESULTS

### 9p21–23 allelic analysis

**Figure 2:**
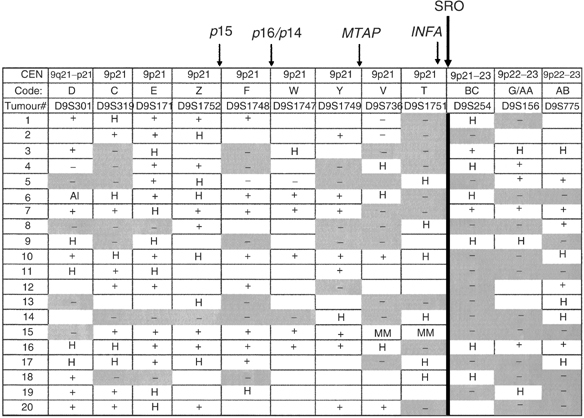
Shortest region of overlap (SRO) at 9p21–23 between markers cent – D9S1751 and D9S254 – pTer, a region telomeric to the CDKN2A and MTAP genes. Nine additional tumours, displaying LOH at a region centromeric to CDKN2A, are not shown. Yellow boxes (−)=loss of heterozygosity. White boxes (+)=retained heterozygosity. (H)=noninformative. (MM)=microsatellite mutation. (AI)=allelic imbalance from a triploid tumour. Blank boxes=not tested.

LOH defined as loss of two contiguous microsatellite markers was detected in 29 (16.4%) of the 177 tumours in the first screening. This proportion was higher (22.6%) in favourable nonstage 4 (4s and LR) than in stage 4 tumours (11.7%). Of 29, 20 (68.9%) demonstrated an SRO between markers D9S1751 and D9S254, a region telomeric to the *CDKN2A* and *MTAP* genes and near the *IFN* gene cluster (Figure 2

). This region was the most commonly deleted in both the favourable (64%) and unfavourable (83.3%) stages. A second region with frequent LOH occurred in 9/29 (31%) tumours and was centromeric to the *CDKN2A* locus (D9S1748).

### Clinical outcome and 9p21–23 LOH

**Figure 3:**
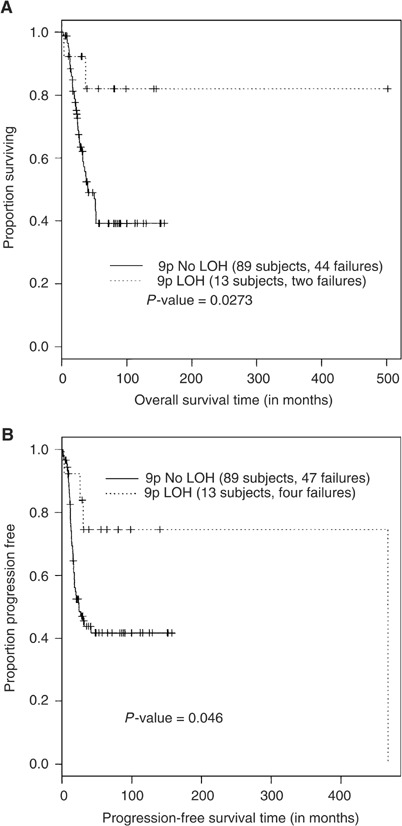
Stratification of overall (**A**) and progression-free (**B**) survival according to the 9p21 allelic status, using the method of Kaplan and Meier. LOH=loss of heterozygosity.

A significantly better overall and progression-free survival was detected in stage 4 NB patients with LOH at the SRO (*P*=0.0273 and 0.046, respectively) (Figure 3

). In total, 80% of stage 4 patients with 9p21–23 LOH were alive 150 months after diagnosis *vs* 40% patients with retained heterozygosity. For nonstage 4 subgroups (LR and stage 4s) no survival differences could be found (*P*>0.05).

### Correlation between clinical and genetic variables for stage 4 patients

Significant correlation was found between age and histology; elevated LDH and unfavourable histology; LDH and MYCN amplification; elevated ferritin and MYCN amplification, gain of material at 17q and intact 11q 23 region. MYCN amplification statistically correlated with 1p36 LOH as reported, and 1p36 LOH correlated with loss of material at chromosome 1p22 as previously reported (Mora *et al*, 2000).

Sex, age at diagnosis, LDH, ferritin, histopathology, MYCN, ploidy and allelotype for chromosomal arms 1p36, 1p22, 11q23, 14q32, 19q13, 17q and 9p21 were analysed for potential prognostic value (Table 1A

**Table 1:** Univariate and multivariate analyses of survival

		***P*-value**
(A) Univariate analysis of survival		
	9p21 LOH	0.044
	11q23 LOH	0.0398
	LDH	0.0768
	MYCN	0.0901

(B) Results of the multivariate analysis (Cox models)		
Model 1	LDH	0.034
*N*=89	9p21 LOH	0.066
		
	LDH+9p21 LOH	0.0181
	likelihood ratio	8.03
		
Model 2	MYCN	0.059
*N*=101	9p21 LOH	0.051
		
	MYCN+9p21 LOH	0.0131
	likelihood ratio	8.67

Only variables with *P*-values equal or less than 0.1 are listed.

). Among all the biologic and clinical features studied only 9p21 and 11q23 LOH were identified as potential risk factors that indicated superior chances of OS (*P*=0.044 and 0.0398, respectively). Patients with 9p21 or 11q23 LOH had significantly better survival than patients with an intact 9p21 or 11q23 regions. MYCN amplification and elevated LDH showed marginally significant *P*-values (0.0901 and 0.0768, respectively) associated with poor OS (see Mora *et al*, 2002b for further information).

Cox proportional hazard models were applied for multivariate analyses of potentially predictive factors, including the four markers that showed best *P*-values associated with OS in the univariate analysis described above. The best modal combinations of paired variables showing independence predicting OS are shown in Table 1B. The models LDH+9p21 and MYCN+9p21 LOH showed significance as predictive factors for poor outcome. When one global, multivariate test, with all four markers associated with poor OS was analysed, we found that none of the variables remained independent and the combined *P*-value was not significant (0.0544).

### Analysis of CDKN2A, CDKN2B and MTAP

**Figure 4:**
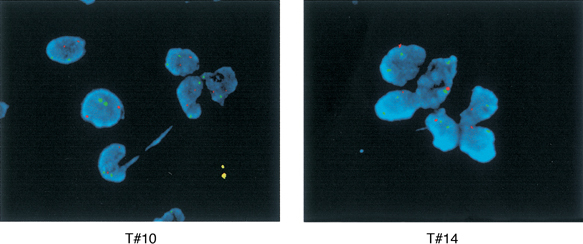
FISH analysis performed on one case with retained heterozygosity at 9p21–23 (tumour #10) and one case with LOH and loss of one of the copies of the gene CDKN2A/p16^INK4a^ (tumour #14). Chromosome 9 centromeric probe (green signals) as well as a locus-specific probe for CDKN2A/p16^INK4a^, CDKN2A/p14^ARF^ and CDKN2B/p15^INK4b^ (orange signals) were used.

Hemizygous deletions of *CDKN2A* and *CDKN2B* were detected by FISH with probe P1 clone 1063 in two of the 21 tumours available for study (9.5%) (Figure 4

). They were both stage 4 patients: case #14 (Figure 2), with extensive allelic loss of 9p and a tumour displaying LOH at a region centromeric to the *CDKN2A* locus (data not shown) (markers D9S1752 and D9S1748). Analysis of *MTAP* exons in tumours with 9p21–23 LOH did not detect deletions or sequence mutations. Only one (10%) of the 10 NB with LOH at the SRO analysed by immunohistochemistry displayed decreased expression of MTAP protein compared to endothelial cells in the same sample. This was a stage 4 NB tumour (case #2) in which LOH was detected for microsatellites D9S319, D9S171 and D9S1752, a region centromeric to the *MTAP* gene. All other tumours had immuno-detectable levels of MTAP protein expression.

Analysis of genome-wide expression profiles by oligonucleotide microarrays in tumours with 9p21–23 LOH and with normal 9p21–23 status. Diminished expression of 363 mRNAs was found in tumours displaying 9p21–23 LOH. Only five of them were encoded by genes located at 9p. Four probe sets were ESTs encoded by genes mapping to 9p24.1 (GenBank Accession #AI084974), 9p24.2 (GenBank Accession #H15396 and #AI917470) and 9p13.2 (GenBank Accession #AA278423), respectively. Only one of the probe sets corresponded to a characterised gene, *nuclear factor IB* (GenBank Accession #U70862), located on 9p22.3, and included in the SRO, according to the Ensembl Genome Browser. No significant change was detected for *CDKN2A/*p16^INK4a^, *CDKN2A/*p14^ARF^_,_ *CDKN2B*/p15^INK4b^, *MTAP*, *IFNA* and *IFNB* mRNAs in tumours with 9p21–23 LOH when compared to those with retained heterozygosity.

## DISCUSSION

Our results show that 9p21–23 LOH is an infrequent event in NB that usually occurs in a region telomeric to 9p21. LOH at this region is more frequently detected in favourable NB stages and is statistically associated with a better clinical outcome in stage 4 patients.

We observed that 9p21–23 LOH occurs at a low frequency in NB tumours (16.4%), similar to results obtained by Marshall *et al* (1997), who reported 9p21 LOH in 17% of NB analysed. In another study of patients identified by a mass screening programme, 9p21 LOH was more frequently detected, although this cohort was likely to include a higher proportion of low-risk tumours (Takita *et al*, 1997).

Approximately 70% of tumours in our series displaying 9p21–23 LOH had a SRO at a region telomeric to the *CDKN2A* and *MTAP* genes, near the *IFNA* and *IFNB* genes cluster. Homozygous or hemizygous deletions of the *α*-, *β*-, and/or *ω*-*IFN* genes have been reported in other malignancies such as acute lymphoblastic leukaemia, and head and neck cancer. Given the statistical association between LOH at the *IFN* gene cluster and recurrence in head and neck cancer, Lydiatt *et al* (1998) suggested the existence of a TSG in this region. However, no specific gene has yet been found and our results add to other data supporting that the region involved in NB biology lies telomeric of the *IFN* gene cluster (Giordani *et al*, 2002).

Previous reports have provided conflicting results on the correlation between LOH at 9p21 and prognosis in NB patients. Takita *et al* (1997) reported that patients with LOH at 9p21 showed statistically significant association with poor prognosis. Conversely, Marshall *et al* (1997) found no correlation between 9p21 LOH and clinical outcome, while others have observed a statistically significant association between LOH at a region telomeric to 9p21 and good prognosis for NB patients (Giordani *et al*, 2002). Our results support the latter study and show that LOH at 9p21–23 is more frequently found in favourable stages of NB. Moreover, LOH at 9p is associated with a significant better overall and progression-free survival in stage 4 patients.

Our results also suggest that *CDKN2A* gene deletions are rare events in NB tumours, in agreement with several prior studies in NB (Beltinger *et al*, 1995; Diccianni *et al*, 1996; Kawamata *et al*, 1996; Castresana *et al*, 1997; Iolascon *et al*, 1998). However, Thompson *et al* (2001) found homozygous deletion of the *CDKN2A* locus in four of 46 NB cell lines analysed and in two of the corresponding primary tumours. They suggested that *CDKN2A* inactivation was an *in vivo* genetic event contributing to tumour biology rather than an *in vitro* phenomenon. However, the incidence of mutations or homozygous deletions has been reported to be lower in primary tumours than in cell lines (Spruck *et al*, 1994) and the possibility of an *in vitro* origin of those deletions cannot be excluded.

Overexpression of *CDKN2A***/**p16^INK4a^ mRNA and protein without genetic alteration of *CDKN2A* has been described in NB (Diccianni *et al*, 1996; Omura-Minamisawa *et al*, 2001). In contrast, Takita *et al* found LOH at the *CDKN2A* locus and lack of p16^INK4a^ expression in cell lines (Takita *et al*, 1997) and in primary tumours (Takita *et al*, 1998). In the latter cohort, p16^INK4a^ immunostaining was undetectable in 61% of patients and this lack of expression correlated with poor prognosis of patients and advance stage of disease (Takita *et al*, 1998). The results we have obtained in a larger series of patients seem to exclude major alterations in the genomic sequence and transcription of genes located on 9p21. Several reasons could account for the disagreement between our results and those reported by other authors. Some of those studies have performed expression analysis on NB cell lines. We have observed that expression profiles of NB cell lines clearly differ from those of their primary tumours (Mora *et al*, 2003). On the other hand, although our results exclude major transcriptional modifications of p16^INK4a^ in primary tumours, we cannot rule out the possibility of translational or post-translational changes that could explain the high proportion of NB with lack of p16^INK4a^ immunostaining in the series reported by Takita *et al* (1998).

To the best of our knowledge, this is the first report examining *MTAP* gene deletions and MTAP protein expression in NB tumours. *MTAP* maps to the 9p21 region and it is frequently codeleted with *CDKN2A*. Deletion of at least one *MTAP* exon was identified in 37.5% of osteosarcomas and *MTAP* mRNA and protein were not detectable in those cases (Garcia-Castellano *et al*, 2002). In our large series of NB, only one sample displayed diminished MTAP immunostaining and no alteration of *MTAP* exons was found. In addition, no significant difference of *MTAP* mRNA expression was detected between NB with 9p21 LOH and those with normal 9p21 status by means of oligonucleotide microarrays.

Finally, given that the more frequently detected SRO included the *IFN* gene cluster, we also compared the expression of *IFNA* and *IFNB* mRNAs in NB with and without 9p21–23 LOH, but did not detect any significant difference between the two groups.

Among the 363 mRNAs that were significantly downregulated in NB displaying 9p21–23 LOH, four were derived from genes mapping to 9p22–24. Only one probe set matched a well-characterised transcript, the human *NFI-B3* mRNA, located on 9p22.3. Nuclear factor I proteins constitute a family of dimeric DNA-binding proteins that function as cellular transcription factors and as replication factors for adenovirus (Qian *et al*, 1995). NFI-B3 is a naturally truncated isoform that includes the DNA binding and dimerisation domains also present in the other NFI family members, although experimental evidences suggest that it cannot bind to DNA by itself. NFI-B3 apparently forms heterodimers with other NFI proteins thereby interfering with their function and is thus considered a transcriptional repressor (Liu *et al*, 1997). Further studies are necessary to investigate the target genes of this repressor activity and to elucidate which of those genes are not repressed in stage 4 NB with LOH at this region and good clinical outcome, as a consequence of the diminished presence of NFI-B3 protein. Their function could shed light on the biological events responsible for the different response to treatment and clinical evolution of stage 4 NB patients.

In summary, our results seem to exclude 9p21 as a critical region in NB biology and point to the existence of potentially deleted genes on 9p22–23. Given the statistical association between 9p21 and 23 LOH with favourable NB stages and stage 4 patients with better clinical outcome, those genes are expected to be involved in biological features related to aggressiveness of NB.
